# Metabolic classifications of renal cell carcinoma reveal intrinsic connections with clinical and immune characteristics

**DOI:** 10.1186/s12967-023-03978-y

**Published:** 2023-02-24

**Authors:** Le Li, Zheng Chao, Un Waikeong, Jun Xiao, Yue Ge, Yanan Wang, Zezhong Xiong, Sheng Ma, Zhihua Wang, Zhiquan Hu, Xing Zeng

**Affiliations:** Department of Urology, Tongji Hospital, Tongji Medical College, Huazhong University of Science and Technology, No.1095, Jiefang Rd, Wuhan, China

**Keywords:** Tumor metabolism, RCC, Metabolic classification, Tumor immunity, Prognosis model

## Abstract

**Background:**

Kidney cancer undergoes a dramatic metabolic shift and has demonstrated responsiveness to immunotherapeutic intervention. However, metabolic classification and the associations between metabolic alterations and immune infiltration in Renal cell carcinoma still remain elucidative.

**Methods:**

Unsupervised consensus clustering was conducted on the TCGA cohorts for metabolic classification. GESA, mRNAsi, prognosis, clinical features, mutation load, immune infiltration and differentially expressed gene differences among different clusters were compared. The prognosis model and nomograms were constructed based on metabolic gene signatures and verified using external ICGC datasets. Immunohistochemical results from Human Protein Atlas database and Tongji hospital were used to validate gene expression levels in normal tissues and tumor samples. CCK8, apoptosis analysis, qPCR, subcutaneously implanted murine models and flowcytometry analysis were applied to investigate the roles of ACAA2 in tumor progression and anti-tumor immunity.

**Results:**

Renal cell carcinoma was classified into 3 metabolic subclusters and the subcluster with low metabolic profiles displayed the poorest prognosis, highest invasiveness and AJCC grade, enhanced immune infiltration but suppressive immunophenotypes. ACAA2, ACAT1, ASRGL1, AKR1B10, ABCC2, ANGPTL4 were identified to construct the 6 gene-signature prognosis model and verified both internally and externally with ICGC cohorts. ACAA2 was demonstrated as a tumor suppressor and was associated with higher immune infiltration and elevated PD-1 expression of CD8^+^ T cells.

**Conclusions:**

Our research proposed a new metabolic classification method for RCC and revealed intrinsic associations between metabolic phenotypes and immune profiles. The identified gene signatures might serve as key factors bridging tumor metabolism and tumor immunity and warrant further in-depth investigations.

**Supplementary Information:**

The online version contains supplementary material available at 10.1186/s12967-023-03978-y.

## Introduction

Metabolic reprogramming of cancer cells is required for tumor genesis and development. Cancer cells autonomously alter their fluxes through a variety of metabolic pathways to meet increased bioenergy and biosynthesis requirements and to alleviate oxidative stress required for cancer cell proliferation and survival [1]. Metabolic phenotypes are frequently defined as an array of intracellular metabolic fluxes supported by the metabolic networks that connect gene–protein–metabolic reactions [2]. Investigations of cancer metabolic phenotypes are receiving growing concerns as it potentially represents a promising therapeutic target. However, awareness that the metabolic phenotype of cancer cells is heterogeneous among varieties of cancers is increasing [3, 4]. In non-small cell lung cancer (NSCLC), cell growth rates do not indispensably rely on the average Lac/Glc ratio, suggesting the Warburg effect is therefore not a universal feature in NSCLC [5]. In hepatocellular carcinoma (HCC), hypermetabolic subclusters displayed low α-fetoprotein (AFP) expression, and good prognosis while subclusters with intermediate metabolic activity displayed high AFP expression level and bad prognosis [6].

Renal cell carcinoma is characterized by disrupted hypoxia-inducible factor (HIF) signaling and perturbed cellular metabolism [7]. Different genetic mutations caused distinct metabolic-oriented variations and histological classifications of RCC [8]. A well-known metabolic hallmark of clear cell renal cell carcinoma (ccRCC) is an aberrant accumulation of lipid and glycogen [9], which has been reported recently as a secondary and dispensable consequence of active HIF-1α signaling and disruption of glycogen synthesis did not affect tumor growth in murine models [10]. Lipid metabolic reprogramming, however, is believed to get involved in a series of micro and macro-level life activities including cellular energy homeostasis, biofilm synthesis, lipid signal transduction, and phenotypic transformation in RCC [11].

It remains unclear whether different clinical stages and outcomes are driven by specific metabolic phenotypes of RCC. Aggressive types of RCC demonstrated distinct metabolic patterns including downregulation of the TCA cycle and decreased AMPK and PTEN protein levels [12]. Men also observed a positive association between tumor progression and metastasis and increased metabolites in glutathione and cysteine/methionine metabolism pathways in glutathione and cysteine/methionine metabolism pathways in ccRCC [13]. In addition, reprogrammed metabolism-induced cellular genome and epigenetic alterations of tumor cells as well as reconstruction of the TME are also accompanied by extensive remodeling of the immune microenvironment [14].

Although kidney cancer is frequently referred to as a kind of “hot tumor” [15], which means abundant lymphocyte infiltration and susceptibility to immunotherapy, the relatively unique and complex immune microenvironment of RCC did pose challenges for treatment options and prognosis evaluation. For example, CD8^+^ cytotoxic T lymphocytes are highly infiltrated in major renal masses but correlate with poorer prognosis [16], conflicting with the recognized knowledge that higher proportions of CD8^+^ T cells prolong patients’ survival. Potential causes include unrecognized tumor-associated antigens [17], T cell exhaustion or anergy [18], metabolic dysregulation of T cells [19] and depleted PBRM1 mutations [20]. However, whether RCC could be classified into several metabolic subclusters and the associations between metabolic phenotypes and immune characteristics as well as prognosis remains unknown up to date. In this study, we sought to objectively classify RCC into distinct metabolic phenotypes and to investigate the clinical and immunological implications, which could inspire the development of new therapeutic solutions to RCC.

## Materials and methods

### Data collection and processing

Patients’ RNA sequencing data, mutation data and corresponding clinical follow-up information were downloaded from the publicly available Cancer Genome Atlas (TCGA) database (https://portal.gdc.cancer.gov). For external validation, the data were downloaded from International Cancer Genome Consortium (ICGC) database (https://dcc.icgc.org/). The downloaded 882 kidney tumor sample count files were loaded into the DESeq2 R package for normalization and differential expression analysis.

### Metabolic genes sources

Metabolism-related pathways were from the GSEA Molecular Signatures Database v7.4 (http://www.gsea-msigdb.org/gsea/msigdb/index.jsp). 2590 metabolic genes from 75 metabolic pathways were selected for further analysis.

### Consensus clustering analysis

The ssGSEA scores of every single sample were assessed using the R package GSVA based on the metabolic dataset expression profiles. Then, unsupervised clustering was conducted based on the ssGSEA scores using the R package ConsensusClusterPlus to group kidney cancer into different metabolic subclusters (50 iterations and 80% resampling rate Pearson correlation).

### Gene-set enrichment analysis

Gene expression data was input into GSEA4.10 software (Gene set database: c2.cp.kegg.v7.4.symbols.gmt) to obtain normalized enrichment scores, *P* values and FDR-q values. |NES|> 1, NOM p-val < 0.05, FDR q-val < 0.25 were set as the cut off values.

### Cancer stem cell index evaluation

As described by Malta et al. [21], the mRNA stemness index (mRNAsi) of each sample was computed using one-class logistic regression machine learning (OCLR) machine-learning algorithm. Then, the mRNAsi for different KIRC metabolic subclusters were calculated.

### Differential expression analysis and multiscale embedded gene co-expression network analysis (MEGENA)

Differential expression analysis was conducted using R package limma with the cut-off |log2foldchange|> 1, padj < 0.05. MEGENA was constructed according to the following steps: (1) correlation assessment by computing Pearson correlation coefficients; (2) construction of fast planar filtered network (PFN) with cut-off FDR < 0.05; (3) multi-scale clustering analysis (MCA).

### Prognostic model construction and validation

The TCGA patients were randomly separated into the training set and test set (7:3 ratio) and the ICGC cohorts were treated as external validation datasets. In the training set and test set, whether the candidate genes of cancer are related to the survival of patients is assessed, key genes are selected, and a risk model is constructed based on the formula: Risk score = h0(t)*exp(β1X1 + β2X2 + … + βnXn). The risk model is verified in the verification set.

### Immunotherapy and chemotherapy analysis

Spearman's correlation was employed to analyze the correlation between tumor immune cell infiltration and prognostic risk score. 198 GDSC (Genomics of Drug Sensitivity in Cancer) drugs for kidney cancer were chosen for analyzing the AUC values in high- or low-risk groups. Lower values of AUC are associated with a higher sensitivity to certain chemotherapy and vice versa.

### Renal cancer cell lines and transfection

Murine renal cancer cell lines Renca were purchased from Procell Life Science&Technology (Wuhan, China). Transfection of ACAA2-V101 plasmids was performed using Lipo3000 (Invitrogen, Carlsbad, CA, USA) following recommended protocol.

### Cell proliferation assay and apoptosis assay

Cell proliferation assay and apoptosis assay were performed as previously described [22]. In short, Renca cells after transfection with ACAA2-V101 and control V101 plasmids were examined for proliferation and apoptosis using corresponding kits.

### Human samples

Resected human renal cell carcinoma tissues were obtained from patients at the Tongji Hospital (Wuhan). Ethical permission was granted by the Clinical Trial Ethics Committee of Tongji Hospital (Wuhan). All patients provided written informed consent to participate in the study.

### Animals

Balb/c mice, 6 to 8 weeks old, were purchased from Cyagen Corporations. These animals were maintained in the Animal Facilities of Tongji hospital experimental animal center under pathogen-free conditions. All studies involving mice were approved by the Animal Care and Use Committee of Tongji Hospital.

### Statistics

All the data were statistically analyzed using Stata version 12.1(Stata Corp.) and R software (version 3.5.2). *P*-value < 0.05 was considered statistically significant.

## Results

### Classification of renal cell carcinoma based on 3 metabolic phenotypes

**Fig. 1:**
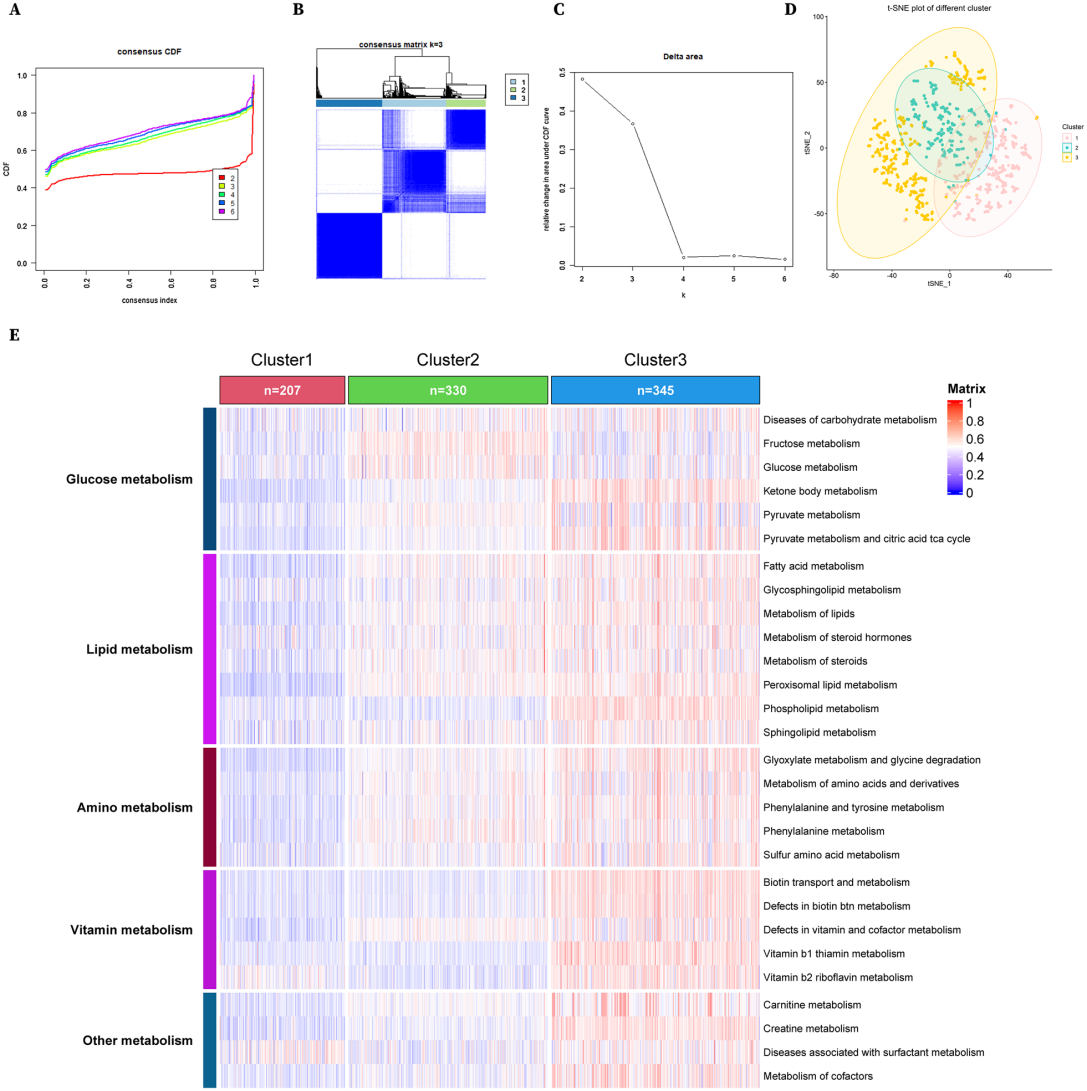
RCC was classified into 3 metabolic subclusters. **A** Consensus clustering cumulative distribution function (CDF) curves. **B** Consensus clustering matrix. **C** Delta area under CDF curves. **D** t-Distributed Stochastic Neighbor Embedding (tSNE) plots of 3 clusters of RCC. **E** Heatmaps of 3 metabolic subclusters of RCC based on ssGSEA scores

Based on the gene expression levels included in 76 KEGG metabolism-related pathways in 882 RCC patients of TCGA, we scored single-sample gene set enrichment analysis (ssGSEA) and made consensus clustering analysis on the scores (Fig. 1A–C). Consequentially, we classified these patients into 3 clusters with low (Cluster 1), medium (Cluster 2) and high (Cluster 3) metabolic phenotypes (Fig. 1D, E). Specifically, Cluster 1 exhibits a hypometabolism pattern in multiple metabolic pathways including fatty acid metabolism, glyoxylate metabolism and glycine degradation, metabolism of lipids compared to both Cluster 2 and Cluster 3 (Fig. 1E and Additional file 1: Figure S1), while Cluster 2 was enriched mainly in cancer and cytokines pathways compared to Cluster 3 (Additional file 1: Figure S1).

### RCC patients with low metabolic phenotypes experienced worse prognosis and higher invasiveness

**Fig. 2:**
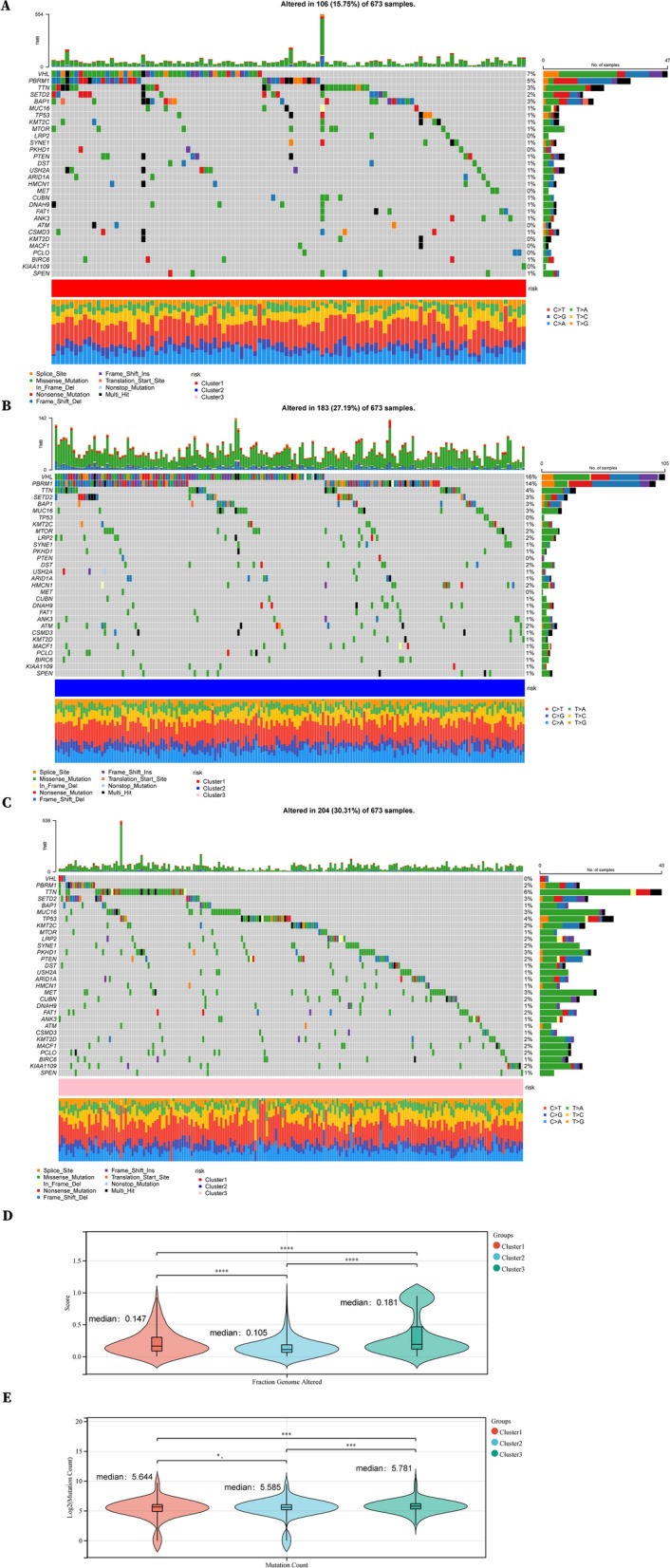
Mutation burden of 3 metabolic subclusters in RCC. **A** Waterfall map illustrating somatic mutation in Cluster 1 and **B** Cluster 2 and **C** Cluster 3. **D** Scores of Fraction Genome Altered and **E** mutation counts in 3 metabolic clusters

To gain a better comprehensive understanding of other characteristics of the 3 subtypes of RCC, we first analyzed the mutation burden for patients in different subtypes. In general, Cluster 3 displayed the highest mutation burden and altered faction genome frequencies while Cluster 1 does the opposite (Fig. 2A–E). Specifically, Cluster 2 displayed high frequencies of VHL and PBRM1 mutation (Fig. 2B). The mutation sites in Cluster 1 were relatively focused (Fig. 2A) while randomly distributed in Cluster 3 (Fig. 2C).

**Fig. 3:**
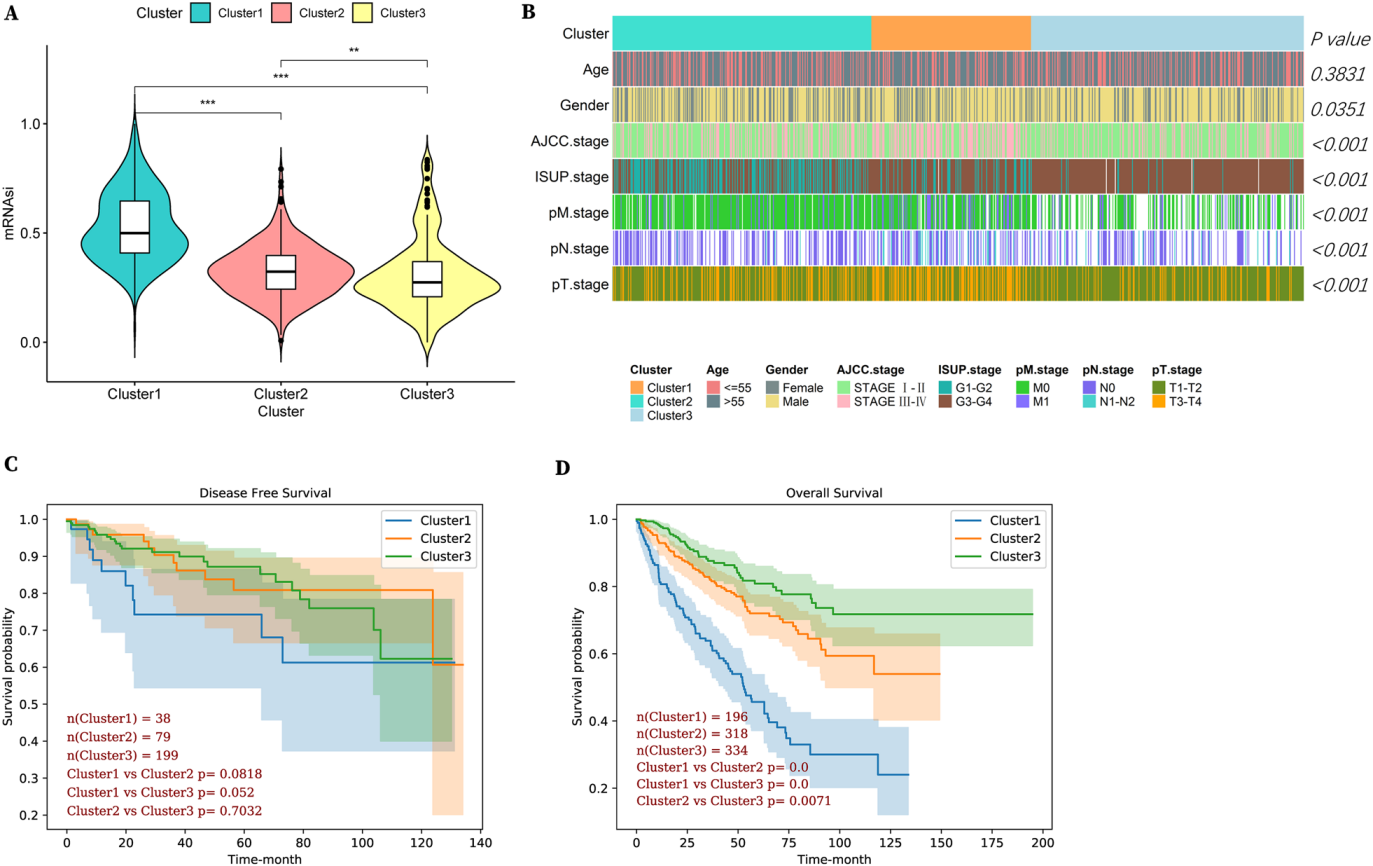
Cluster 1 with low metabolic phenotypes displayed the highest invasiveness and poorest prognosis. **A** mRNAsi scores of 3 subclusters in RCC. **B** Heatmaps illustrating clinical characteristics including age, gender, AJCC stage, ISUP stage, and pTNM stage in 3 subclusters. **C** Disease-free survival and **D** Overall survival in 3 subclusters

Next, we compared among the three clusters the mRNAsi, an index quantifying the levels of cancer stem cells in tumors, and found higher mRNAsi of Cluster 1 (Fig. 3A), implying worse prognosis, more aggressive phenotype and metastasis of this cluster in RCC. Moreover, patients in Cluster 1 experienced the highest pathologic AJCC grade and clinical pM and pT grade (Fig. 3B), further supporting defining Cluster 1 as a “Highly invasive” subcluster. Although no significant survival disadvantage of disease-free survival was shown for Cluster 1 (Fig. 3C), men did observe this trend and significant survival discrepancies in overall survival among the three clusters (Fig. 3D). Collectively, our results reveal low metabolic phenotypes reversely correlate with tumor grade and patient outcome in RCC.

### Low metabolic tumor patterns drive enhanced immune infiltration and immunosuppressive phenotypes in RCC

**Fig. 4:**
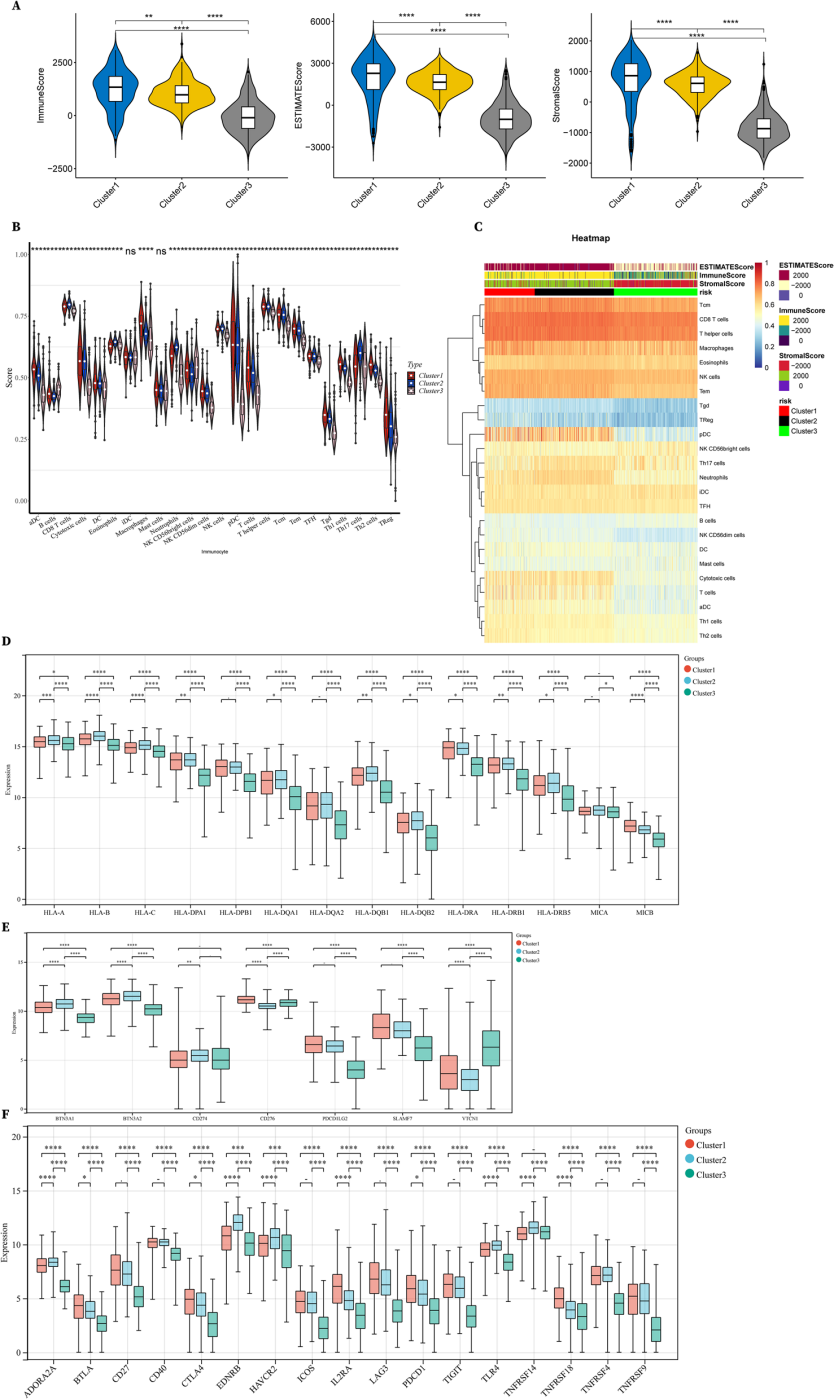
Cluster 1 with low metabolic phenotypes displayed a high-infiltrating but suppressive immune microenvironment. **A** Immunesocres, Estimatescores, and Stromalscores of 3 subclusters. **B** Percentage abundance of 22 types of tumor-infiltrating immune cells in 3 subclusters of RCC. **C** Heatmaps showing immune cell abundance, ESTIMATE, immune and stromal scores of 3 subclusters based on the ssGSEA and ESTIMATE algorithms. **D** Expression of MHC-I molecules in 3 subclusters. **E**, **F** Expression of co-stimulatory and co-inhibitory molecules in 3 subclusters

In order to investigate the immune characteristics of these three clusters, we used both Estimate and ssGSEA algorithms to analyze the differential immune infiltration and expression of immune-associated genes. Counterintuitively, Cluster 1 had maximum immune scores and stromal scores while Cluster 3 had minimum scores (Fig. 4A, C). Specifically, aDCs, macrophages, NK CD56^dim^ cells, Tems, Th1 cells, Th2 cells and Tregs showed significantly higher infiltrating proportions in Cluster 1 compared to the other 2 subclusters (Fig. 4B, C). Analogously, it has been reported in earlier integrated proteogenomic results that higher CD8^+^ T cell infiltration correlates with poorer prognosis in RCC [23], but the underlying mechanisms still remain unclear. Despite that, one observation is noteworthy in our results however, the proportions of tumor-infiltrating macrophages, Th2 cells and Tregs both showed a significant proportion advantage in Cluster 1 with the poorest prognosis compared to those in Cluster 2 or Cluster 3, implying that Cluster 1 could exist as a bilateral immune-infiltration high and immune inhibiting subcluster. To further validate our hypothesis, we analyzed immuno-related gene expression and unsurprisingly found MHC-I-associated genes were highly expressed in Cluster 1 (Fig. 4D), which were positively correlated with immune infiltration in usual cases [24]. Considering the fact that the MHC–peptide complex-mediated first signal, as well as the co-stimulatory second signal, were both required for the activation of anti-tumor immunity, we next explored the expression levels of co-stimulatory/inhibitory molecules. Although the inhibitory ligands and co-stimulatory receptors did not show apparent differences in Cluster 1 compared to those in Cluster 2, co-inhibitory receptors of tumor-infiltrating immune cells were significantly highly expressed in Cluster 1 compared to those in Cluster 2 or Cluster 3, including BTLA, CTLA4, IL2RA, PDCD1 and so on (Fig. 4E, F), suggesting the existence of infiltration high but immune-suppressive tumor microenvironment in RCC.

### Prognosis model construction based on the intersection of DEGs and MEGENA modules

**Fig. 5:**
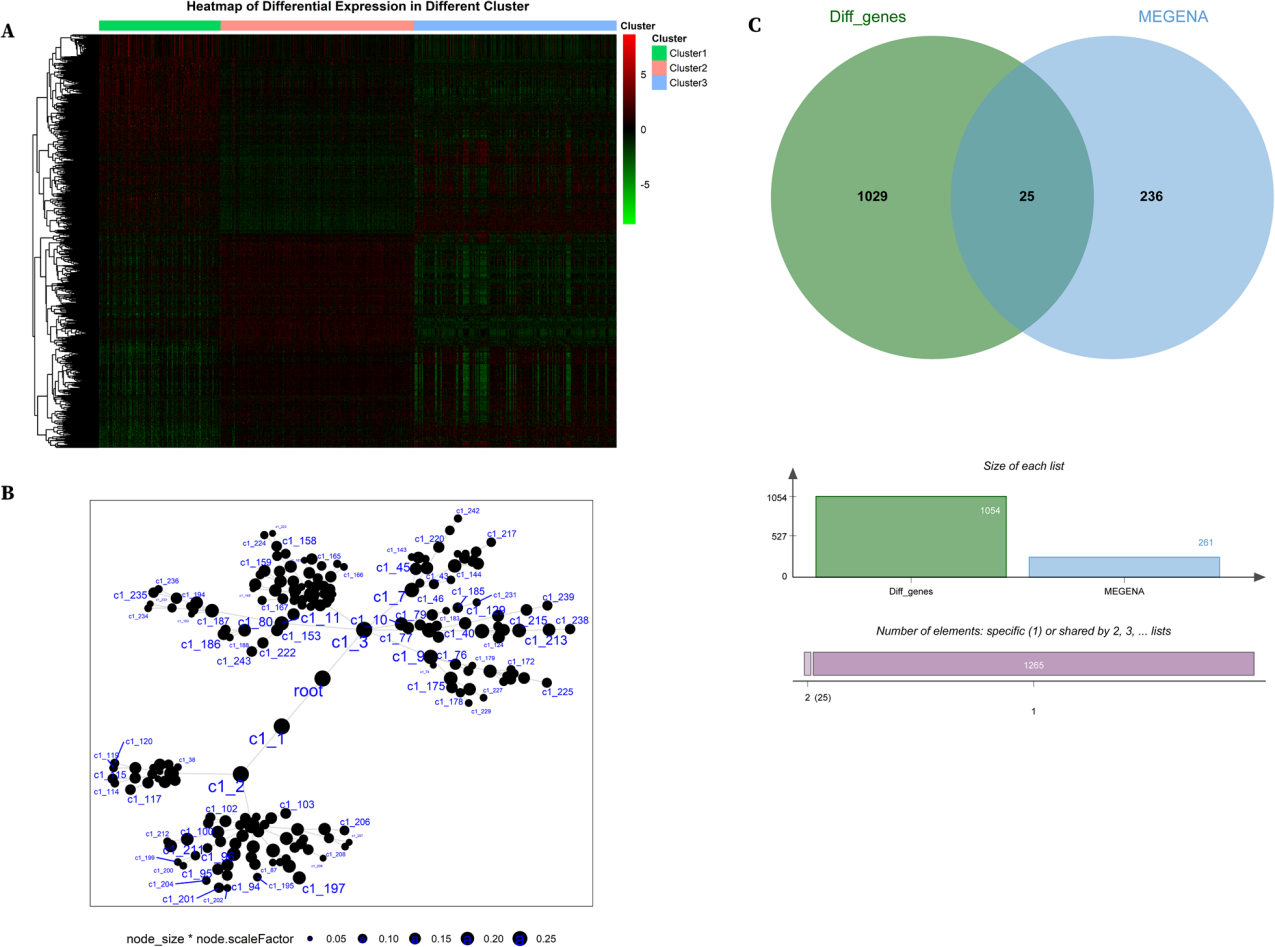
Intersections of DEGs in 3 subclusters and MEGENA of metabolic modules in RCC. **A** Heatmaps of differentially expressed genes (DEGs) in 3 subclusters. **B** Representative illustration of hierarchical clustering modules of metabolic genes in RCC. **C** Venn diagram showing the intersection results of DEGs hub genes and MEGENA genes

To further explore the differences among the metabolic subclusters of RCC and identify critical metabolic genes capable of predicting patients' prognosis, we first conducted differential expression analysis for the three clusters and identified 1054 differentially expressed genes (Fig. 5A). Then, we identified 243 co-expression modules in the metabolic gene network of RCC using the hierarchical clustering method MEGENA (Fig. 5B and Additional file 2: Table S1). By taking the intersection of these two results, we obtained 25 candidate metabolic genes as potential OS predictors (Fig. 5C).

**Fig. 6:**
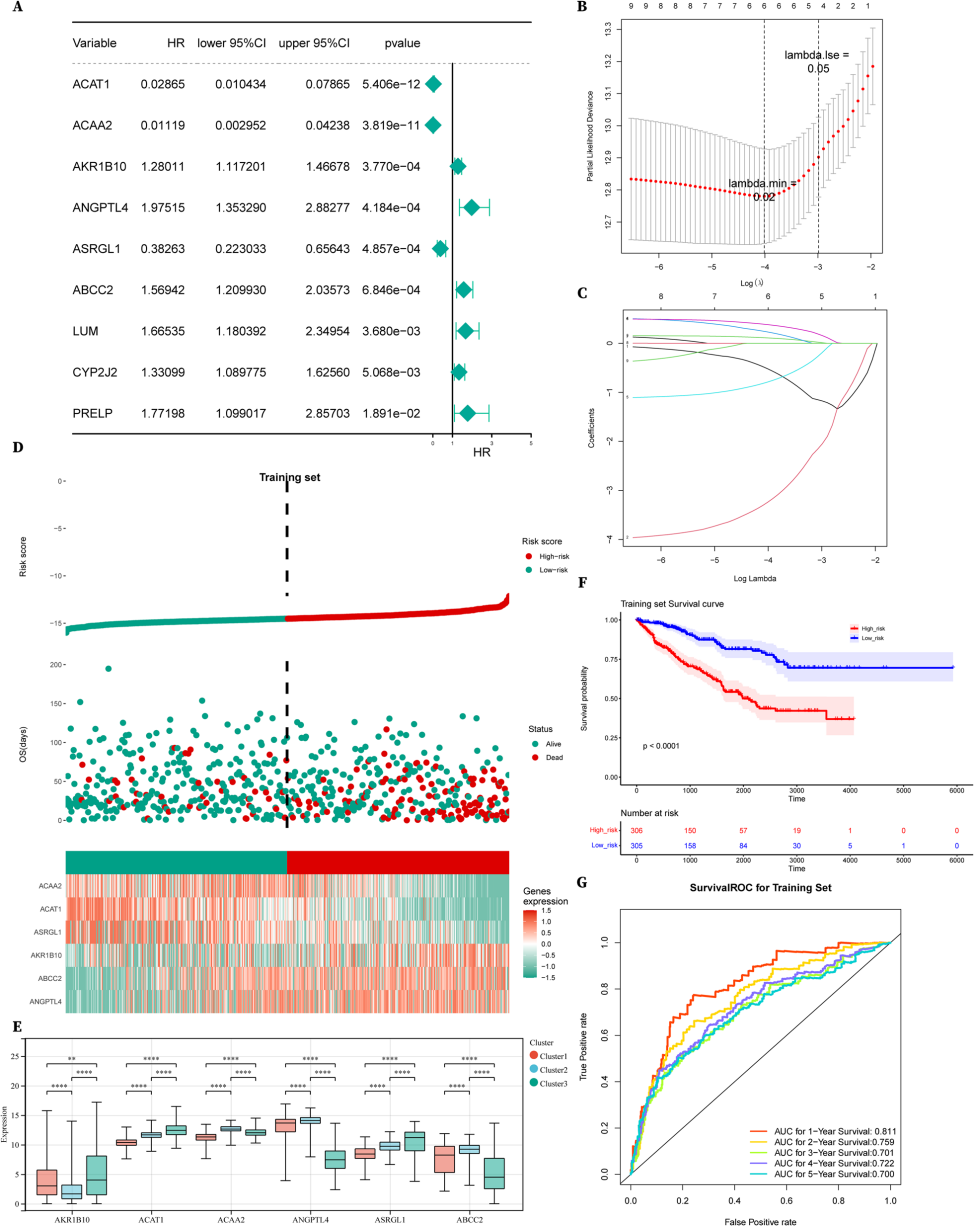
Construction of 6-gene signature prognosis model. **A** Forest plot of Univariate COX analysis. **B**, **C** Coefficient estimates and cross-validation error of the Lasso regression results. **D** Risk curve, survival scatter plot analysis and heat maps of expression profiles of 6 metabolic genes of patients with high- and low-risk in the training set of the 6-gene signature prognosis model. **E** mRNA expression levels of the 6 metabolic genes in 3 subclusters. **F** Kaplan–Meier curve shows the overall survival of high- and low-risk RCC patients. **G** Time-dependent receiver operating characteristic (ROC) curve analysis exhibits the prognostic performance of the 6-gene-signature-based prognostic model in predicting 1 ~ 5-year survival times of the training group

All the 873 RCC samples of TCGA were randomly assigned at a 7:3 ratio to a training set (611 samples) and a test set (262 samples), and 222 samples of ICGC were used as the external validation dataset. To effectively screen these genes, univariate Cox proportional hazards regression analysis was employed with a cut-off *P* value < 0.05 and nine prognosis-associated genes were obtained (Fig. 6A). For further filtering, lasso regression analysis was applied (Fig. 6B, C), identifying 6 genes for prognosis model construction: ACAA2, ACAT1, ASRGL1, AKR1B10, ABCC2 and ANGPTL4.

Next, we used these 6 genes to construct the prognosis model for RCC patients based on the following formula: Risk score = h0(t)*exp (β1X1 + β2X2 + … + βnXn). According to the median risk score, patients were divided into high‐ and low‐risk groups in the training set, test set and validation set respectively. In the training set, AKR1B10, ABCC2 and ANGPTL4 showed high expression in the high-risk group (HR > 1) while ACAA2, ACAT1 and ASRGL1 showed high expression in the low-risk group (HR < 1) (Fig. 6D). Consistent with our previous results, the protective factors ACAA2, ACAT1 and ASRGL1 showed low expression in Cluster 1, the low-metabolic subcluster with the worst prognosis in RCC (Fig. 6E). KM survival curve was plotted to appraise the discrepancy of survival between the high- and low-risk group (Fig. 6F). For the prediction of OS, the 1–5-year AUC values of the ROC curve were all no lower than 0.7, indicating a good survival prediction performance (Fig. 6G).

**Fig. 7:**
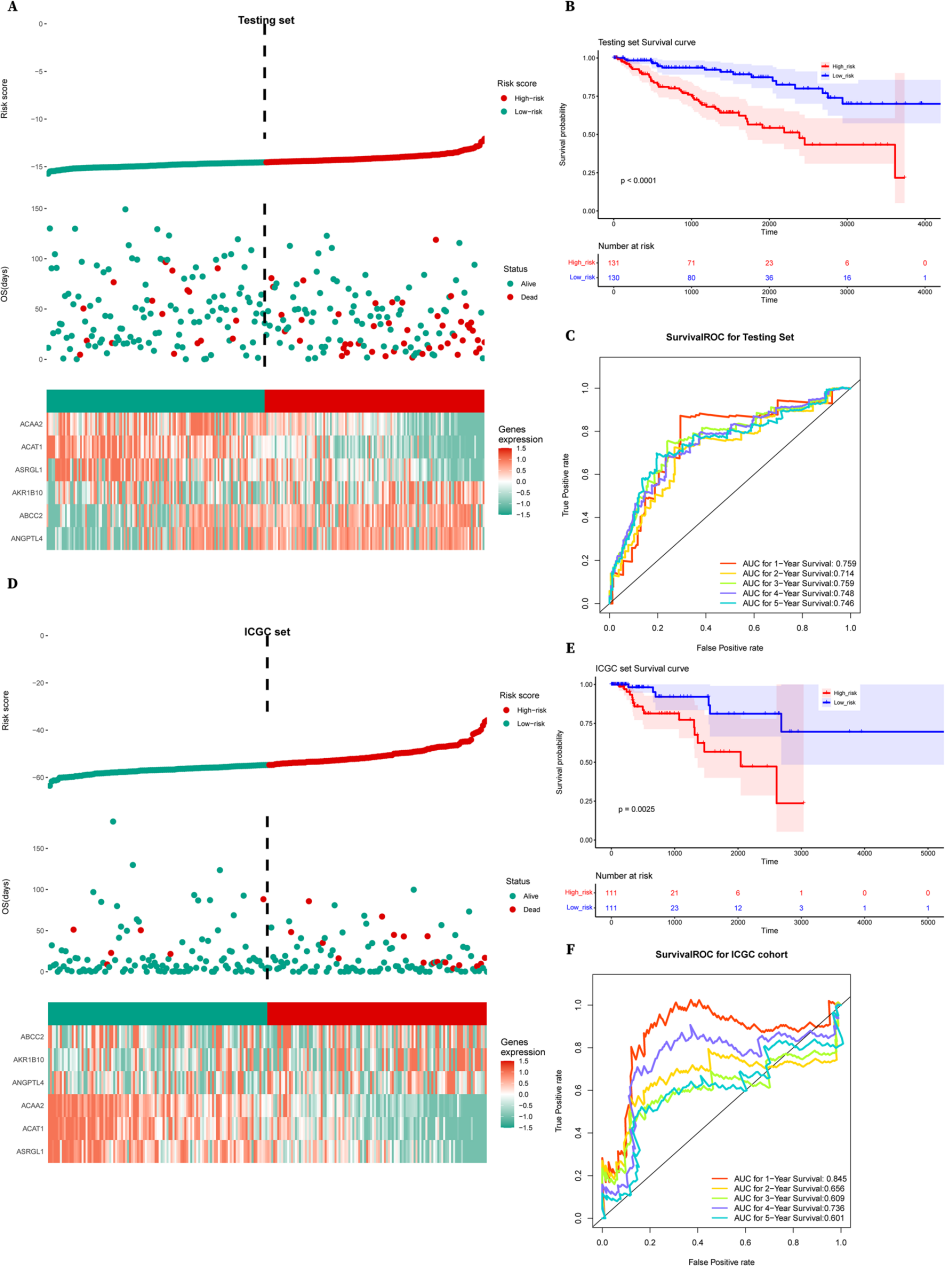
Risk score analysis of the 6-metabolic-gene-signature related prognostic model in the test group and ICGC cohort. **A** Risk curve, survival scatter plot analysis and heat maps of expression profiles of 6 metabolic genes of patients with high- and low-risk in the testing set of the 6-gene signature prognosis model. **B** Kaplan–Meier curve shows the overall survival of high- and low-risk RCC patients in the testing set. **C** Time-dependent (ROC) curve analysis exhibits the prognostic performance of the 6-gene-signature-based prognostic model in predicting 1 ~ 5-year survival times of the testing set. **D** Risk curve, survival scatter plot analysis and heat maps of expression profiles of 6 metabolic genes of patients with high- and low-risk of the 6-gene-signature prognosis model in the ICGC RCC cohort. **E** Kaplan–Meier curve shows the overall survival of high- and low-risk RCC patients in the ICGC RCC cohort. **F** Time-dependent ROC curve analysis exhibits the prognostic performance of the 6-gene-signature-based prognostic model in predicting 1 ~ 5-year survival times of the ICGC RCC cohort

Finally, we performed a similar analysis in the test set and the external validation set and both yielded the same conclusion for the 6 genes (Fig. 7A–F). Importantly, the 1–5-year AUC values of the ROC curve were all higher than 0.6 (Fig. 7C, F), demonstrating the widespread application potential of this prognosis model.

### Nomogram establishment based on the multivariable Cox regression model

**Fig. 8:**
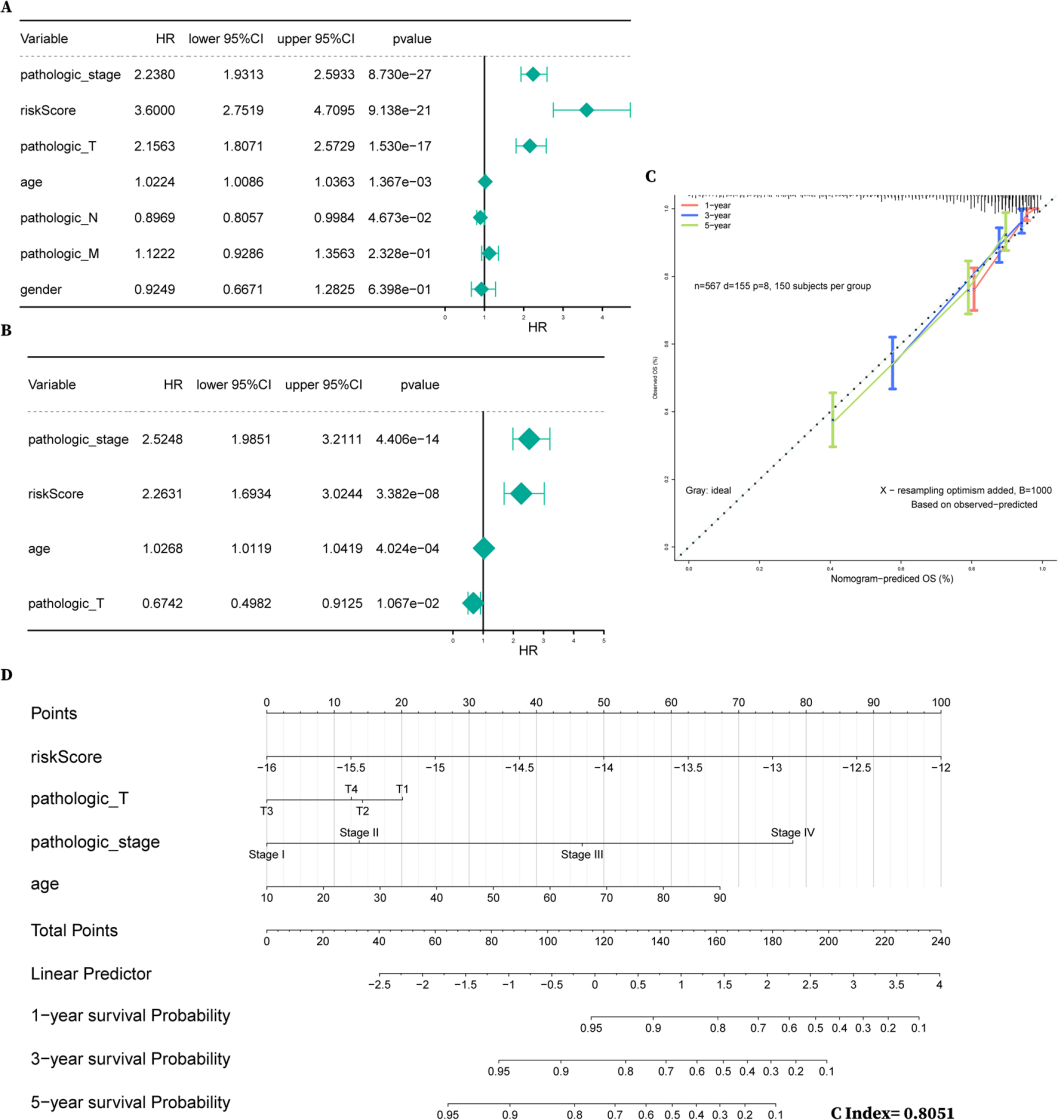
The nomogram based on the 6 metabolic genes for predicting the 1-year, 3-year and 5-year OS of RCC patients. **A** Forest plot of risk scores and clinical features based on univariate Cox regression analysis. **B** Forest plot of significant factors based on multivariate Cox regression analysis. **C** Calibration curves for nomograms predicting 1-year, 3-year and 5-year OS of RCC patients. **D** Nomograms predicting 1-year, 3-year and 5-year OS of RCC patients

We included clinicopathological features such as age, gender, pathologic TNM and pathologic stage, as well as risk score as independent prognostic factors in univariate Cox analysis and found *P* values lower than 0.05 for risk score, pathologic TN, pathologic stage and age (Fig. 8A and Additional file 3: Table S2). According to the results of univariate analysis, we put these indicators into the multivariate Cox model (Fig. 8B). Then, the nomograms and 1–5 year calibration curves were constructed based on the independent prognostic factors with *P* less than 0.05 identified by the multivariable Cox model (Fig. 8C, D). The ultimate predictors of the nomograms included risk score, pathologic T, pathologic stage and age, and the C index was 0.8051, indicating that the nomogram prediction model had good discrimination ability.

### High-risk scores correlate with elevated inhibitory immune checkpoints

**Fig. 9:**
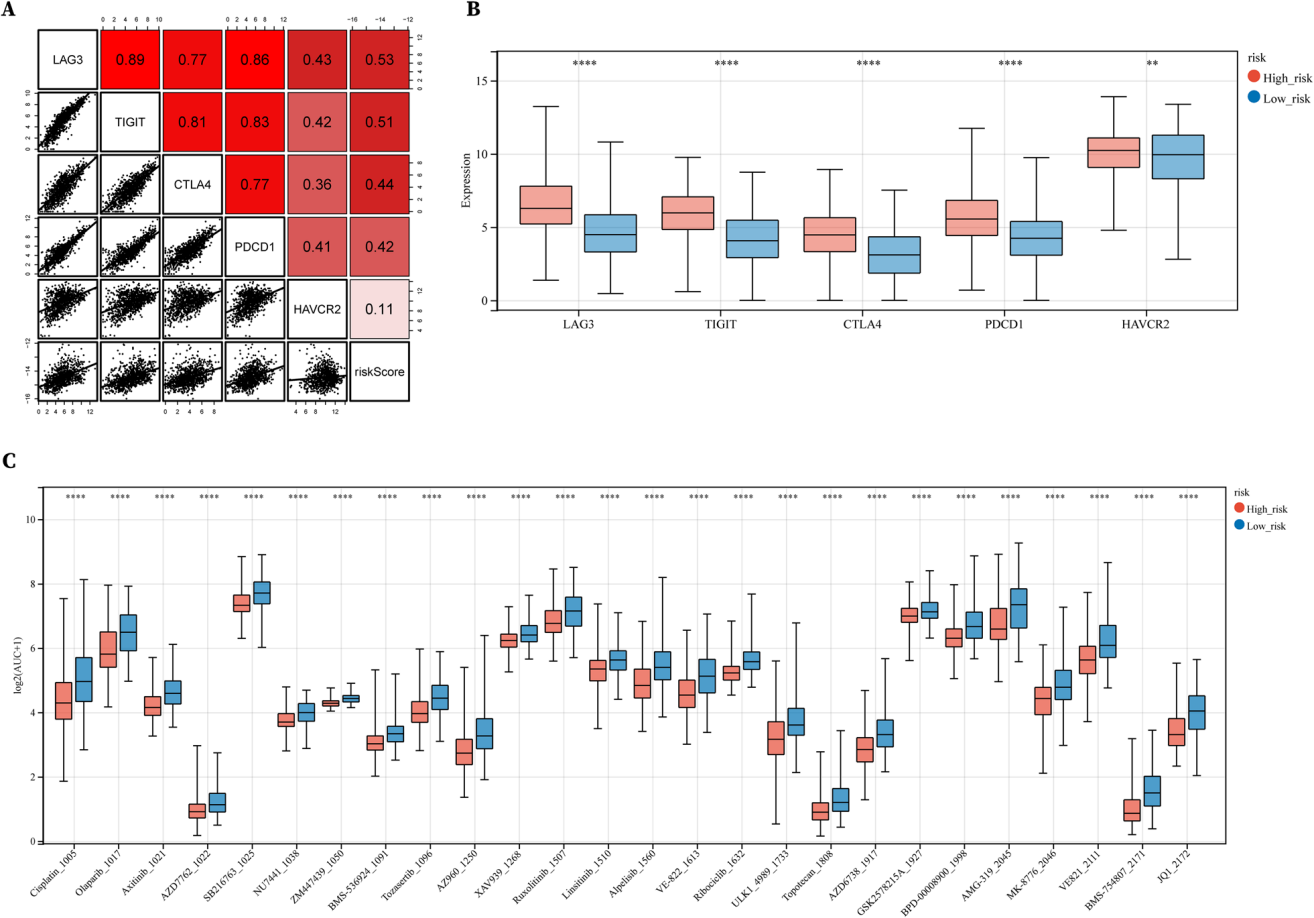
High-risk patients showed high expression of immune-checkpoint molecules and sensitivity to chemotherapies. **A** Correlation of risk scores and expression of immune-check points molecules. **B** Expression of immune-checkpoint molecules in low- and high-risk patients. **C** Sensitivity to chemotherapies of low- and high-risk patients. Low AUC values indicate higher sensitivity to chemotherapies

Since the patients with high-risk scores had poorer survival and we observed in the previous Cluster 1 that higher expression levels of inhibitory immune checkpoint molecules were positively correlated with poorer survival, we continued to investigate their expression in high- and low-risk groups. Consistently, the expression of immune checkpoint molecules including LAG3, TIGIT, CTLA4, PDCD1 and HAVCR2 were all highly expressed in the high-risk group compared to those in the low-risk group (Fig. 9A, B). We also investigated potential chemo‐sensitive drugs for the high-risk group and found 26 drugs with negative correlation coefficients, including Olaparib, Axitinib, Cisplatin and Ruxolitinib (Fig. 9C), suggesting the promising anti-tumor effects of a single drug or combination treatment with ICBs for high-risk patients of RCC.

### Diagnostic value assessment for the prognostic model

**Fig. 10:**
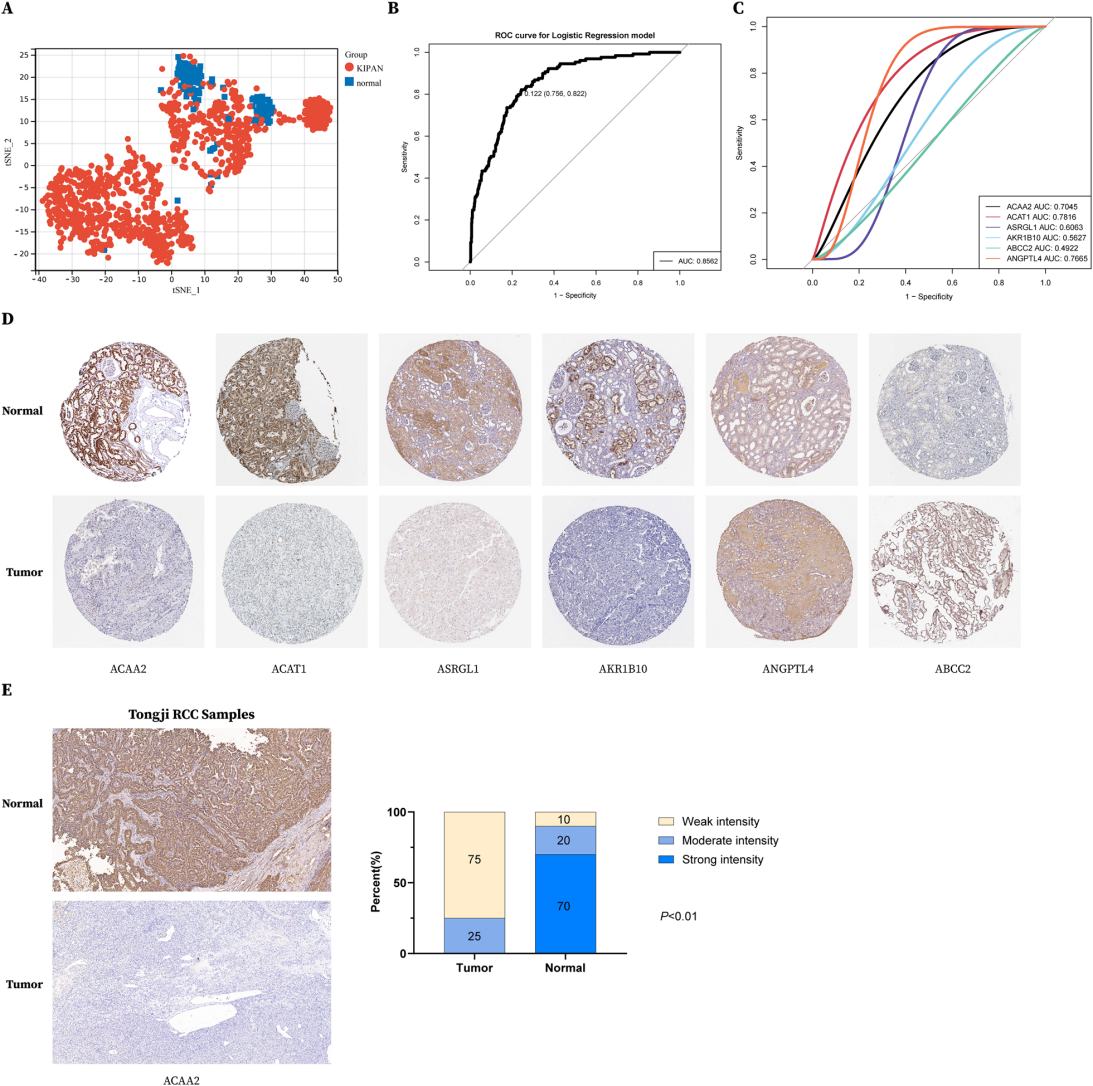
Investigations of diagnostic values of 6 metabolic genes. **A** t-SNE clustering results of the 6 metabolic genes in distinguishing RCC tumor and normal samples. **B** ROC curve for the 6-gene signature logistic regression model. **C** ROC curve for the independent 6 metabolic genes. **D** IHC results indicating expression of ACAA2, ACAT1 and ANGPTL4 in RCC tumor samples and normal samples from the Human Protein Atlas database. **E** Representative (left) and statistical analysis (right) of IHC results indicating expression of ACAA2 in RCC tumor (n = 20) and normal tissues (n = 20) from Tongji hospital

Based on the 6 biomarkers in the prognostic model, we wondered about their capacity in distinguishing RCC patients from control normal samples in TCGA. Using tSNE analysis for the expression levels of the six biomarkers, we found cancer patients were well distinguished from normal patients (Fig. 10A). What’s more, the AUC value of the ROC curve for the logistic regression model was 0.856, indicating an excellent diagnostic power (Fig. 10B). As for independent predictors, the AUC value of ACAA2, ACAT1 and ANGPTL4 were 0.7045, 0.7816 and 0.7665, respectively (Fig. 10C). Using IHC data from the Protein atlas database, we observed strong or moderate staining for ACAA2, ACAT1, ASRGL1, and AKR1B10, weak staining for ANGPTL4 and ABCC2 in normal kidney tissues and opposite staining results in kidney tumor samples(Fig. 10D). Moreover, we collected 20 RCC tumor samples and 20 adjacent normal samples and observed 75% weak intensity in tumor samples and 90% strong or moderate intensity in normal samples for ACAA2 expression using immunohistochemical analysis (Fig. 10E), revealing its potential anti-tumor role in RCC.

### ACAA2 overexpression delays tumor growth and increases tumor immune infiltration

**Fig. 11:**
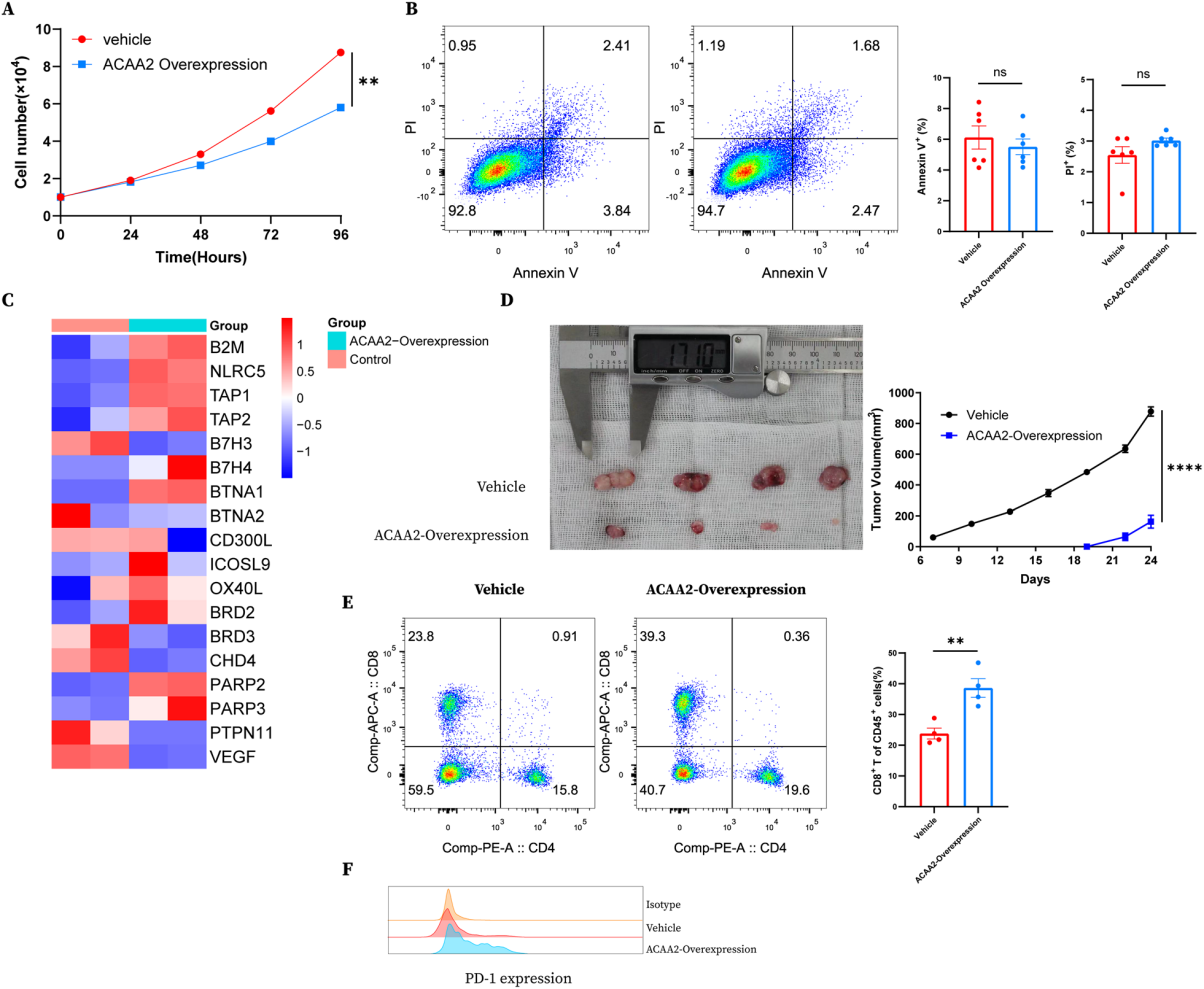
ACAA2 overexpression inhibits tumor growth in vitro and in vivo and positively correlates with CD8^+^ T cell immune infiltration. **A** CCK8 results showing Renca cell proliferation after transfection with control and ACAA2-V101 plasmids. **B** Representative and statistical results showing the apoptotic changes of Renca cells after transfection with control and ACAA2-V101 plasmids for 48 h. **C** qPCR results of gene expression changes of Renca cells after transfection with control and ACAA2-V101 plasmids for 48 h. **D** Tumor volumes of Balb/c murine models subcutaneously transplanted with Renca cells after transfection with control and ACAA2-V101 plasmids. **E** Representative and statistical results showing tumor infiltrating CD8^+^ T cells in the vehicle group and ACAA2-Overexpression group. **F** PD-1 expression of CD8^+^ T cells in the vehicle group and ACAA2-Overexpression group

To further investigate the potential roles of ACAA2 in renal cell carcinoma, we constructed the ACAA2-V101 plasmids and transfected them into renca cells. Surprisingly, the cells transfected with ACAA2-V101 plasmids showed significant growth inhibition compared to those transfected with control vehicle plasmids (Fig. 11A), while no difference in apoptotic changes was observed (Fig. 11B). Next, we conducted qPCR analysis on 786O cells transfected with ACAA2-V101 plasmids and found upregulation of MHC-I associated genes like B2M, NLRC5, TAP1 and TAP2 (Fig. 11C). In subcutaneously implanted Balb/c murine model, the ACAA2-Overexpression group showed significant tumor growth delay compared to the vehicle group (Fig. 11D). Moreover, tumor-infiltrating CD8^+^ T cells and PD-1 positive CD8^+^ T cells proportions were higher in the ACAA2-Overexpression group (Fig. 11E, F), implying that ACAA2 might serve as a promising immunotherapeutic target in RCC.

## Discussion

The currently widely accepted classification method of RCC is based on the heterogeneous histopathologic characteristics of RCC. However, different subtypes of RCC also share some commonalities including high infiltrating but inhibitory immune microenvironment, acquired metabolic disorders like aberrant glycolysis, intermediate mutation load and so on [25]. Our research thus grouped kidney cancer and classified them into 3 metabolic subclusters based on unsupervised clustering, attempting to provide new insights into the diagnosis and treatment for RCC.

In our findings, kidney cancer was classified into high-, medium- and low-metabolic subclusters and the subcluster with low metabolic phenotypes displayed the poorest prognosis, strongest tumor stemness and aggressiveness, intermediate mutation load and highest immune infiltration. For the counter-intuitive results of high infiltration and poor prognosis in RCC, one plausible explanation is that memory T cells are retained in a quiescent or exhausted state. It has been reported that high expression profiling of the inhibitory receptors LAG-3, TIM-3, and TIGIT in renal cell carcinoma refers to malignancy and decreased survival [26, 27]. Other factors potentially contributing to the inhibitory tumor microenvironment include increased Tregs, neutrophil and monocyte infiltration [14]. In addition, newly published research indicated that elevated expression of the type 2 T helper cell signature also promoted maintenance of the suppressive immune microenvironment and was correlated with poorer survival in ccRCC, pRCC and chRCC [8].

In order to identify critical genes contributing to the formation of distinct metabolic patterns in RCC, we took the intersection of the DEGs among the three clusters and MEGENA results of significantly clustering metabolic genes in RCC. After strict screening, 6 metabolic genes were identified: ACAA2, ACAT1, ASRGL1, AKR1B10, ABCC2 and ANGPTL4. Based on these metabolic gene signatures, we constructed a prognosis model and verified the predicting capacity both internally and externally using the ICGC RCC database. Moreover, we further included clinical factors with significant prognostic values and successfully constructed a nomogram with a C index of 0.8015, indicating a relatively good discriminative ability.

In our prognosis model, 3 genes including AKR1B10, ASRGL1 and ABCC2 showed relatively poorer predictive power by a single gene. AKR1B10 encodes a member of the aldo–keto reductase (AKR) 1B subfamily and exerts variable or even opposing roles in different tumor contexts [28, 29]. ASRGL1 encodes the enzyme that catalyzes the hydrolysis of l -asparagine to l-aspartic acid and ammonia and plays a protective role in endometrial carcinoma [30]. ABCC2, ATP Binding Cassette Subfamily C Member 2, acts as a key transporter in support of cell energy transition and proliferation and was identified as a pro-oncogenic factor in colorectal cancer [31]. The other 3 genes in the prognosis model associated with lipid metabolism regulation displayed the diagnostic potential to discriminate between normal and kidney tumor tissues: ACAT1, ANGPTL4 and ACAA2. ACAT1 is a key cholesterol esterification enzyme mediating an aerobic process breaking down fatty acids into acetyl-CoA [32]. Pharmacological inhibition or genetic ablation caused an increase in the plasma membrane cholesterol level of CD8^+^ T cells and thus led to potentiated effector function and enhanced proliferation [33]. In tumor cells, however, ACAT1 is "hijacked" to regulate pyruvate dehydrogenase complex (PDC) and support the Warburg effect in human cancer [34]. ANGPTL4 encodes a glycosylated, secreted protein containing a C-terminal fibrinogen domain, which functions as a serum hormone regulating glucose homeostasis and lipid metabolism [35]. The ANGPTL4/NOX4 axis is critical for tumor cell extravasation and metastatic seeding of tumor cells in dyslipidemia-associated cancer like kidney cancer [36], head and neck squamous cell carcinoma [37] and so on. ACAA2 (Acetyl-CoA Acyltransferase 2) catalyzes the last step of the mitochondrial beta-oxidation pathway, and little is known about its roles in tumor genesis and metastasis. The latest research indicated knockdown of ACAA2 increased the proliferation of multiple human HCC cell lines in vitro and accelerated the formation of xenograft tumors in nude mice [38]. Our results revealed the tumor-suppressing role of ACAA2 both in vitro and in vivo in RCC. Furthermore, this anti-tumor effect was also associated with enhanced tumor immune infiltration and might be a promising target in combination with anti-PD-1 therapy.

## Conclusions

In conclusion, we developed a novel metabolic classification method of RCC and fully explored the immunological and clinical characteristics of the three sub-clusters. Furthermore, we identified and validated the tumor-inhibiting roles of ACAA2, which facilitates the development of diagnosis and treatment for RCC patients.

## Supplementary Information

**Additional file 1.** Gsea results of 3 clusters.

**Additional file 2.** Partial profiles of MEGENA-hub modules.

**Additional file 3.** Clinical characteristics of high- and low-risk group in the prognostic model.

## Data Availability

The data that support the findings of this study are available from the corresponding author upon reasonable request.

## Abbreviations

RCC: Renal cell carcinoma
GEO: Gene expression omnibus
GO: Gene ontology
GSEA: Gene set enrichment analysis
ssGSEA: Single sample gene set enrichment analysis
KEGG: Kyoto encyclopedia of genes and genomes
TMB: Tumor mutational burden
FDR: False discovery rate
PD1: Programmed death-1 receptor
CDF: Cumulative distribution function
t-SNE: T-Distributed stochastic neighbor embedding
ROC: Receiver operating characteristic
