# Supplementary figures and images for: Metabolic classifications of renal cell carcinoma reveal intrinsic connections with clinical and immune characteristics
Source: J Transl Med. 2023 Feb 24;21:146. doi: 10.1186/s12967-023-03978-y (PMC9960222; doi:10.1186/s12967-023-03978-y)

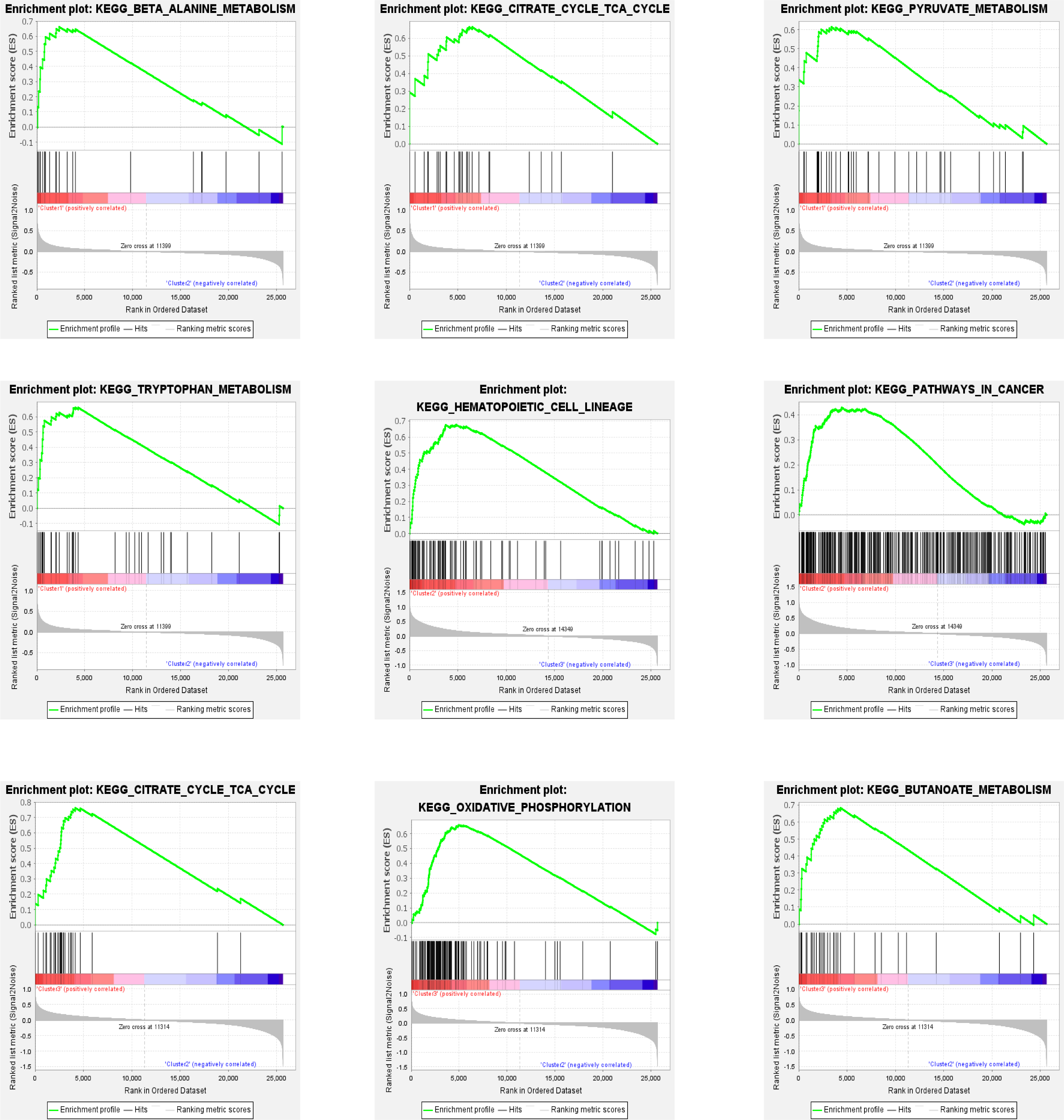

Supplement: Supplementary file 3 — Additional file 3. Clinical characteristics of high- and low-risk group in the prognostic model. [file 12967_2023_3978_MOESM3_ESM.tif]
